# 10 % fluorescein sodium vs 1 % isosulfan blue in breast sentinel lymph node biopsy

**DOI:** 10.1186/s12957-016-1031-1

**Published:** 2016-11-03

**Authors:** Lidong Ren, Zhao Liu, Mengdi Liang, Li Wang, Xingli Song, Shui Wang

**Affiliations:** 1 Department of Breast Surgery, The First Affiliated Hospital of Nanjing Medical University, 300 Guangzhou Road, Nanjing, 210029 China; 2 Department of Breast Surgery, Inner Mongolia People’s Hospital, Hohhot, China; 3 Department of Thyroid and Breast Surgery, Affiliated Hospital of Xuzhou Medical College, Xuzhou, China

**Keywords:** Fluorescein sodium, Isosulfan blue, Breast sentinel lymph node biopsy

## Abstract

**Background:**

Sentinel lymph node biopsy (SLNB) is well accepted to be a standard procedure in breast cancer surgery with clinically negative lymph nodes. Isosulfan blue is the first dye approved by the USA Food and Drug Administration for the localization of the lymphatic system. Few alternative tracers have been investigated. In this study, we aimed to compare the differences between 10 % fluorescein sodium and 1 % isosulfan blue in breast sentinel lymph node biopsy and to investigate the feasibility of using 10 % fluorescein sodium as a new dye for breast sentinel lymph node biopsy.

**Methods:**

A total of 30 New Zealand rabbits were randomly divided into the fluorescein sodium group and the isosulfan blue group (15 rabbits per group). Fluorescein sodium or isosulfan blue was injected subcutaneously into the second pair of mammary areolas.

**Results:**

The average fading time of the second lymph nodes in the isosulfan blue group was significantly shorter than that in the fluorescein sodium group. Moreover, the detection rates of SLNs were higher in the fluorescein sodium group than in the isosulfan blue group. No significant differences between the fluorescein sodium group and isosulfan blue group were observed regarding the distances between the detected sentinel lymph nodes and second pair of mammary areolas, the distances between the second lymph nodes and second pair of mammary areolas, the number of detected sentinel lymph nodes and second lymph nodes, the average dyeing time of the sentinel and the second lymph nodes, and the average fading time of the second lymph nodes.

**Conclusions:**

In summary, we first reported that fluorescein sodium is a potential new tracer for breast sentinel lymph node biopsy.

## Background

Breast cancer is the most common carcinoma in females and the second leading cause of cancer death in women [1]. The sentinel lymph node (SLN) status, an accurate predictor of axillary lymph node status, has been shown to be one of the most important prognostic factors in breast cancer [2]., Sentinel lymph node biopsy (SLNB) is a safe and reliable technique that is well accepted as a standard procedure in breast cancer surgery with clinically negative lymph nodes [3].

Currently, different tracer materials have been reported in SLNB of breast cancer, such as blue dye, radioisotope, carbon nanoparticles, or a combination [4, 5]. However, there is no consensus regarding the optimal tracer materials for SLNB [6]. The detection rate of blue dye is lower than that of radioisotope despite its cost effectiveness and safety [4]. The application of radioisotope in SLNB is hampered by the risk of radiation exposure and poor quality of lymph vessel mapping [7, 8]. The use of carbon nanoparticles is limited by the high cost. Therefore, the development of new tracer materials for SLNB is still needed.

Fluorescein sodium (FS) is a fluorescent dye that is widely used in ophthalmic surgery and malignant brain tumor surgery [9, 10]. Ten percent FS, a widely available substance, is conventionally safe without any clearly associated adverse effects. Furthermore, FS was reported to be an effective tracer material for SLN mapping in colorectal tumors [11]. However, to our knowledge, the use of FS in SLNB of breast cancer has not been reported previously. In this study, we aimed to compare the usefulness of 10 % FS and 1 % isosulfan blue (commonly used blue dye) to investigate the feasibility of using 10 % FS in SLNB of breast cancer.

## Methods

### Animal experiments

Thirty female New Zealand White rabbits weighing between 2.0 and 2.5 kg were randomly divided into the FS group and isosulfan blue group (15 rabbits each group).

Solutions of 10 % FS (Santa Cruz Biotechnology, Dallas, TX, USA) and 1 % isosulfan blue (US Surgical Corp, North Haven, CT, USA) were prepared using sterile water for injection as the solvent. Rabbits were anesthetized with pentobarbital intravenously (15 mg/ml, 2 ml/kg, pentobarbital sodium; Sigma, St, Louis, MO, USA). After anesthesia, rabbits were positioned supine and the planned injection sites were clipped. The skin flaps were elevated from the second mammary glands and extended to fossa axillaris bilaterally. Careful blunt dissection was performed to avoid the injury of blood vessels and lymphatic vessels. Intradermal injection of 0.3 ml 10 % FS or 1 % isosulfan blue was made at the bilateral sites of the second mammary gland of rabbits in FS group or isosulfan blue group, respectively.

**Fig. 1:**
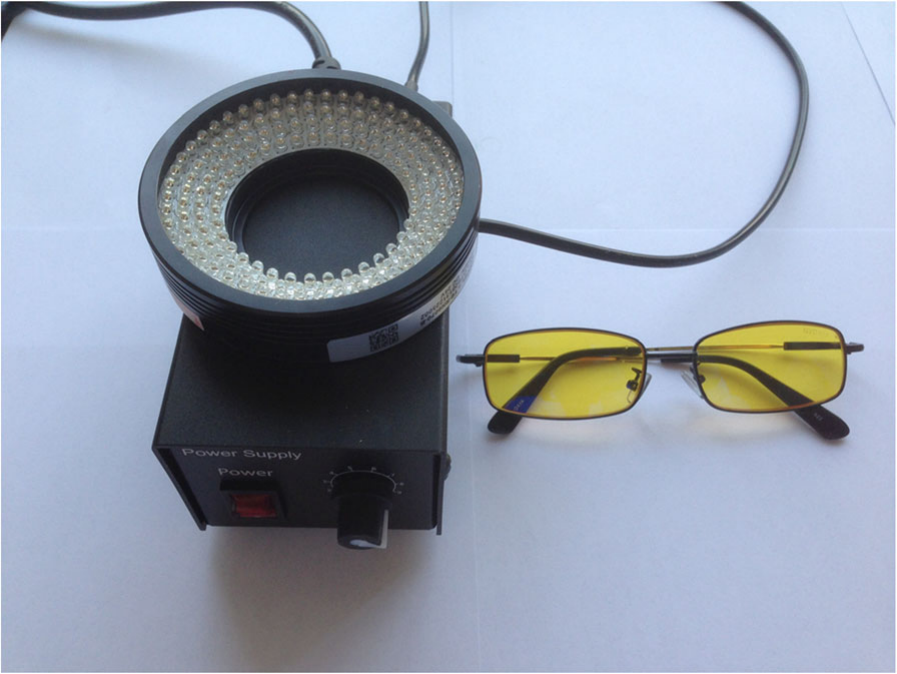
LTS-RN9070 blue light source system and goggle with blue light filter

The operating room lights were adjusted to the dim mode, and a hand-held 485-nm wavelength blue light source (Model: LTS-RN9070; Ling Liang Vision, Shanghai, China) was used as exciting light for FS. Goggles with a blue light filter were equipped to search for the yellow-green FS-detected SLNs (Fig. 1). The FS-detected SLNs were defined as the first one to two fluorescent lymph nodes in the dark background. The FS-detected second lymph nodes were defined as the fluorescent lymph nodes receiving drainage from the FS-detected SLNs and connected with the fluorescent lymphatic vessels. On the other hand, the isosulfan blue-detected lymph nodes were observed under the operating room lights in the regular mode. The isosulfan blue-detected SLNs were defined as the first one to two blue-stained lymph nodes visible in the fossa axillaris area. The isosulfan blue-detected second lymph nodes were blue-stained lymph nodes that received drainage from the isosulfan blue-detected SLNs. The two tracers were compared for the average dyeing time of the sentinel and second lymph nodes, average fading time of the sentinel and second lymph nodes, detection rates, distances between the detected SLNs and second pair of mammary areolas, and distances between the second lymph nodes and second pair of mammary areolas, as well as the number of detected SLNs and second lymph nodes.

After the observation of all of the data, animals were sacrificed with pentobarbital intravenously (325 mg/ml, 10 ml).

### Statistical analysis

The numerical data were presented as the means ± standard deviation (SD), and *p* values were calculated by Student’s *t* test. Fisher’s exact test was used to compare the detection rates of SLNs between the two groups. All of the statistical analyses were carried out using STATA 11 (StataCorp LP, College Station, TX, USA). Differences with a *p* value less than 0.05 were considered statistically significant.

## Results

### General aspects

All rabbits in the FS group and isosulfan blue group were kept in the same cleaning cages and were fed with the same rabbit feed (corn 80 %, hay 10 %, bran 5 %, bone meal 3 %, salt 2 %, no autofluorescence ingredient was included). No significant differences were observed between the FS group and isosulfan blue group regarding weight, the distances between the detected sentinel lymph nodes and second pair of mammary areolas, or the distances between the second lymph nodes and second pair of mammary areolas (Table 1).

**Table 1:** General aspects of animals in two groups

Variable	FS group	IS group	*p* value
Body weight (kg)	2.54 ± 0.17	2.56 ± 0.15	0.57
Distance from SNP to SLN (cm)	11.39 ± 3.14	12.34 ± 1.72	0.23
Distance from SNP to 2^nd^ LN (cm)	15.09 ± 2.86	15.22 ± 1.64	0.89

*FS* fluorescein sodium, *IS* isosulfan blue, *SNP* the second pair of nipples, *SLN* sentinel lymph node, *2* ^*nd*^ the second lymph node

*p < 0.05

### Afferent lymphatic vessels stained by FS and isosulfan blue

**Fig. 2:**
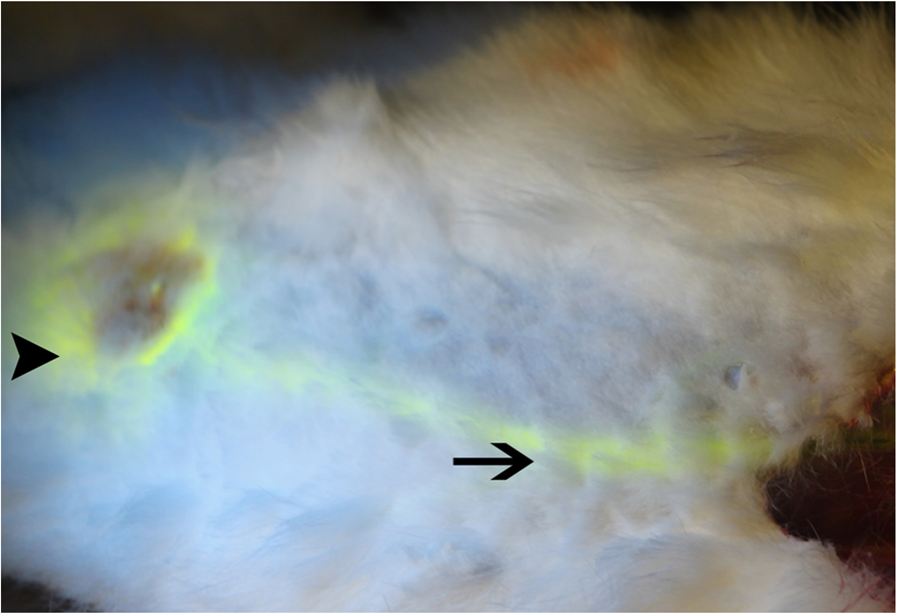
Percutaneously observable FS signal in a lymphatic vessel. The second mammary glands are stained by FS (*arrow head*), and the afferent lymphatic vessel is stained by FS (*arrow*)

**Fig. 3:**
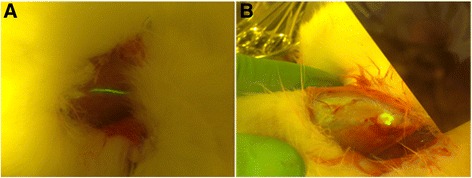
Lymphatic vessel and lymph node stained by FS

The afferent lymphatic vessels stained by FS were easily visualized under florescent light even when they were covered by tissues (Figs. 2 and 3). However, the lymphatic vessels stained with isosulfan blue were faint and difficult to observe under incandescent or fluorescent light. The detection rate of afferent lymphatic vessels using FS was higher than that using isosulfan blue (100 vs 80 %, respectively) (Table 2).

**Table 2:** Comparative analysis of various parameters between two groups

Variable	FS group	IS group	*p* value
Detection rate of ALV	100 % (30/30)	80 % (24/30)	0.024*
Number of the 2^nd^ LN	1.36 ± 0.62	1.13 ± 0.65	0.92
Dyeing time of the SLN (s)	142 ± 69	206 ± 219	0.28
Dyeing time of the 2^nd^ LN (s)	502 ± 417	483 ± 305	0.91
Fading time of the SLN (s)	4327 ± 2359	3482 ± 1680	0.3445
Fading time of the 2^nd^ LN (s)	3918 ± 2196	1332 ± 443	0.0077*
Detection rate of the SLN	100 % (30/30)	80 % (24/30)	0.024*

*FS* fluorescein sodium, *IS* isosulfan blue, *ALV* afferent lymphatic vessels, *SLN* sentinel lymph node, *2* ^*nd*^ the second lymph node

*p < 0.05

### Comparison of FS and isosulfan blue in lymph node dissection

A mean of 1.36 SLNs per side and a mean of 0.88 of the second lymph nodes per side were identified in the FS group. By contrast, the average number of SLNs and second lymph nodes per side identified in the isosulfan blue group was 1.38 and 0.79, respectively. No significant differences were found between the two groups (Table 2).

There were no significant differences between the FS group and isosulfan blue group in the average dyeing time of the SLNs (142 vs 206 s) and second lymph nodes (502 vs 483 s, respectively). Furthermore, no significant differences were observed between the FS group and isosulfan blue group in the fading time of SLNs (4327 vs 3482 s, respectively). However, the fading time of the second lymph nodes was much longer in the FS group than in the isosulfan blue group.

The detection rates of SLNs in the FS group were each 100 %. By contrast, the detection rates of SLNs in the isosulfan blue group (80 %) were significantly lower than those in the FS group (*p* = 0.024). To further investigate the superiority of FS in SLN detection, we performed intradermal injection of 0.3 ml of 10 % FS into the second mammary gland of rabbits in which isosulfan blue was failed to detect the SLNs. All of the SLNs that could not be detected in the isosulfan blue group were successfully identified by the supplementary injection of FS.

### Side effects

No side effects were observed in either the FS group or isosulfan blue group during the process of tracer injection and lymph node dissection.

## Discussion

Isosulfan blue is the first dye to be approved by the USA Food and Drug Administration for the localization of the lymphatic system [12]. Although various tracer materials for SLNB have emerged in the past two decades, isosulfan blue is still accepted as one of the most effective tracers in SLNB because of the simplicity of its use and availability [11]. Despite the wide use of isosulfan blue in SLNB of breast cancer, isosulfan blue has been reported to cause erroneous pulse oximetry readings [13]. Furthermore, the relatively high cost of isosulfan blue has limited its popularization in developing countries.

FS was reported as an alternative tracer with some clear advantages in sentinel node mapping in colorectal tumors. In this study, 1 % isosulfan blue was used as a control tracer to compare the usefulness of FS with that of isosulfan blue and to evaluate the feasibility of using 10 % FS in SLNB of breast cancer.

Isosulfan blue is a reliable tracer material in lymphatic vessel identification during SLNB of breast cancer [14, 15]. However, the detection rate of afferent lymphatic vessels by isosulfan blue is only 80 % in our study. By contrast, all of the afferent lymphatic vessels were identified in the FS group. On the other hand, it may take a long time for surgeons to learn the blue dye method [16]. Our results have shown that the fluorescence in FS-stained lymphatic vessels and lymph nodes could easily penetrate the fur and tissues of rabbits and could be visualized percutaneously. Thus, FS-based SLNB is much easier to perform and requires a shorter learning curve than isosulfan blue.

In our study, the detection rate of SLNs was 80 % for isosulfan blue-guided SLNB, a finding that is consistent with that in other studies showing a detection rate ranging from 75 to 90 % with isosulfan blue dye [17, 18]. By contrast, the identification rate of SLNs in the FS group was 100 %.

Skip metastases are not very common, and the incidence has been reported to be 15 % for breast cancer [19]. However, the presence of skip metastasis and non-sentinel lymph node metastasis did affect the success of SLNB in breast cancer. Therefore, it is essential to evaluate the effectiveness of tracers in identifying second lymph nodes that are considered potential metastatic non-sentinel lymph nodes. In our study, there was no statistically significant difference in the average dyeing time and number of second lymph nodes in the FS group compared with those in the isosulfan blue group. Nevertheless, the fading time of second lymph nodes was much longer in the FS group than in the isosulfan blue group, potentially providing surgeons with additional time to identify the metastatic non-sentinel lymph nodes.

As a fluorescent dye, indocyanine green (ICG) has been regarded as a novel method in SLNB in breast cancer. The detection rate of using ICG was reported to be 80 %, which is slightly lower than that of FS [20]. Furthermore, the use of ICG is associated with high cost equipment [21]. Recently, a new non-radioactive method was developed, using superparamagnetic iron oxide (SPIO) nanoparticles as tracer and manual magnetometer as detector. This new method was reported to be non-inferiority compared to the combination of radioisotope and blue dye. However, the application of SPIO is limited by the high cost and the requirement of special plastic surgical material, while the usage of FS as tracer for SLNB is more economical (5 ml FS: 20 US dollar, goggle: 30 US dollar/pair, blue light source: 400 US dollar).

## Conclusions

In summary, our study is the first report to compare the use of 10 % FS and 1 % isosulfan blue in SLN mapping of breast cancer. We found that it is feasible and safe to use FS in SLNB of breast cancer. Moreover, as a potential tracer, FS is superior to isosulfan blue regarding certain aspects of SLNB of breast cancer. However, our data are based on animal models; further studies are still required to evaluate the safety and feasibility of using FS in SLNB of breast cancer in patients.

## Abbreviations

FS: Fluorescein sodium
SLN: Sentinel lymph node
SLNB: Sentinel lymph node biopsy
